# The Toxic Actions of Urotropin

**Published:** 1903-09-19

**Authors:** 


### THE TOXIC ACTIONS OF UROTROPIN.

Urotropin is an unstable chemical compound formed by the action of formaldehyde on ammonia. It is readily decomposed, with the liberation of free formaldehyde, by weak acids and even acid salts such as the acid sodium phosphate of the urine. The drug given internally is the most reliable urinary antiseptic known. In a very interesting paper read before the New York Academy of Medicine Dr. Warren Coleman[1] put forward the results of an inquiry which he had made into the literature of the subject in order to find out what instances had been reported of urotropin giving rise to untoward symptoms. Nicolaier, who first used urotropin in 1894 as a diuretic and uric acid solvent, and later as a urinary antiseptic, was in the habit of employing doses of from 120 to 150 grains a day, but undesirable symptoms made him reduce this amount considerably; he has never seen any ill effects from daily doses of less than 30 grains. Others have observed toxic effects to follow the administration of 15 grains a day, and Dr. Coleman has had ill results follow a dose of 7½ grains. Individuals vary greatly in their susceptibility; children usually stand urotropin well. In order to lessen the chances of undesirable symptoms following the use of the drug it is necessary to dilute it very freely, so that a dose of 7½ grains, for instance, should be dissolved in at least half a pint of water. When taken by the mouth urotropin is rapidly absorbed, since it begins to appear in the urine within ten minutes of its administration. It is believed to undergo no change while circulating in the blood; though from the clinical evidence of its action in cases of cystitis with ammoniacal urine the extent of the decomposition before the urotropin reaches the bladder must be considerable, because it is not decomposed in alkaline solutions at the body temperature. This latter fact must be borne in mind, since the formaldehyde, on which the antiseptic virtues of urotropin almost certainly depend, is only liberated in acid urine, and therefore drugs which diminish the acidity of the urine should not be given in conjunction with urotropin. Formaldehyde is capable of entering into a loose combination with uric acid, forming a soluble compound, and it was therefore hoped that urotropin would be of benefit in gouty conditions, but these anticipations have not been fulfilled clinically.

The toxic actions of urotropin include (1) irritation of the stomach, (2) diarrhœa and abdominal pain, (3) irritation of the skin with a rash resembling that of measles, (4) headache and ringing in the ears, (5) irritation of the kidneys, (6) strangury, (7) hæmaturia and hæmoglobinuria. Of these, Dr. Coleman states, strangury is the commonest, and in one of his own cases such intense strangury followed a single dose of 7½ grains of urotropin that opium and hyoscyamus suppositories had to be given to relieve the pain. Wider experience with urotropin has shown that vesical inflammation is not an infrequent result of its administration. The symptoms may vary from slight increase of frequency of micturition to intense strangury, accompanied by the presence of blood in the urine. Dr. Coleman finds seven cases of hæmaturia caused by urotropin recorded in the literature, while he has had one case under his own care in which both hæmaturia and hæmoglobinuria followed the taking of 7½ grains of urotropin. Whether the blood in these cases comes from the bladder or the kidneys or both has not yet been decided, though the presence of hæmoglobinuria in Dr. Coleman's case would seem to point to a renal origin. All the untoward symptoms mentioned above are more likely to occur if the drug is not properly diluted. It is interesting to note that the more important of these toxic actions have been produced by intravenous injection of formaldehyde both in rabbits and in the human.

[1] Medical News, Aug. 29th, 1903.

